# Patterns in California Ambulance Patient Offload Times by Local Emergency Medical Services Agency

**DOI:** 10.1001/jamanetworkopen.2024.51022

**Published:** 2024-12-16

**Authors:** Madeline Feldmeier, Karen Patricia Reyes, Crystal Chen, Karl A. Sporer, Zita Konik, Hernando Garzón, Renee Y. Hsia

**Affiliations:** 1 Department of Emergency Medicine, University of California, San Francisco; 2 Alameda County Emergency Medical Services Agency, Alameda, California; 3 California Emergency Medical Services Authority, Rancho Cordova; 4 Philip R. Lee Institute for Health Policy Studies, University of California, San Francisco

## Abstract

**Importance:**

Ambulance offload delays are a timely and crucial issue with implications for patients, emergency medical services (EMS) agencies, hospitals, and communities. Published data on recent patterns in ambulance patient offload times (APOTs) are sparse.

**Objective:**

To examine patterns in APOT by California local EMS agency and variation between and within local agencies.

**Design, Setting, and Participants:**

This cohort study used APOT reports from the California EMS Authority and local EMS agencies between January 1, 2021, and June 30, 2023, to examine patterns in ambulance offload times. County-level population data were collected from the US Census Bureau to calculate mean annual offloads per 1000 population.

**Exposure:**

Ambulance transport to emergency departments.

**Main Outcomes and Measures:**

Ambulance offload volumes, mean annual offloads per 1000 population, APOT-1 (a reporting metric that includes the 90th percentile ambulance offload time and number of offloads to a specific hospital) weighted means (SDs), and APOT-1 medians (IQRs).

**Results:**

A total of 5 913 399 offloads across 34 California local EMS agencies were analyzed. The APOT-1 weighted mean (SD) across the state was 42.8 (27.3) minutes, and the median (IQR) monthly hospital-level APOT-1 was 28.9 (14.9-46.3) minutes. Nearly one-half of local EMS agencies (16 of 34 [47.1%], accounting for 79.2% of all offloads) experienced an APOT-1 weighted mean greater than the 30-minute standard set by the state. Moreover, 20 of 33 local EMS agencies (60.6%) reported an annual APOT-1 weighted mean that was worse in 2023 than 2021.

**Conclusions and Relevance:**

In this cohort study, approximately half of all agencies consistently reported ambulance offload times greater than the 30-minute state standard, and there was significant variation between and within agencies. These findings may spur collaborative efforts between stakeholders to determine the most effective strategies for addressing systemic issues resulting in long APOT across California.

## Key Points

**Question:**

What are the most recent patterns in patient offload times for ambulances transporting patients to emergency departments in California?

**Findings:**

In this cohort study of 5 913 399 offloads across 34 local emergency medical services (EMS) agencies, the mean ambulance offload time across the state was 42.8 minutes, and the median monthly offload time by hospital was 28.9 minutes. Nearly half of all local EMS agencies (16 of 34) had a mean offload time greater than the 30-minute standard set by the state.

**Meaning:**

These findings are particularly concerning given that patients rely on and expect a rapid response from ambulance services, and offload delays decrease ambulance availability for other patients in the vicinity.

## Teaser

This cohort study examines patterns of ambulance patient offload time in California by local emergency medical services (EMS) agency and variations between and within agencies.

## Introduction

There has been increasing attention paid to rising response times for emergencies requiring ambulance transport. Central to this issue are long ambulance patient offload times (APOTs), a crucial interval marking the duration from an ambulance’s arrival at the emergency department (ED) to the point where the patient is formally transferred to the ED’s care.^1^ More commonly known as wall time, APOT has garnered widespread attention globally^2,3^ and locally in the US,^4,5^ particularly as EDs continue to face long wait times and chronic crowding and boarding.^6,7,8,9,10^

Empirical evidence has shown that the implications of long ambulance offload times extend beyond the inconvenience of delays in patient care. Offload delays are associated with an increased length of stay in the ED^11,12^ and increased patient mortality.^2,12^ An ambulance stuck at the wall means decreased availability of critical care services in the community and increased ambulance response times for other patients in the vicinity who may require ambulance transport.^13,14,15,16,17,18^ It also means a loss of ambulance unit hours (each hour of ambulance service) for emergency medical services (EMS) systems (eg, in California, Riverside and San Bernardino Counties logged 20 535 total delay hours in 2012, resulting in $3 million in lost unit hours^14^). In light of these sobering implications, offload delays remain a persistent problem and are ultimately a symptom of larger system-level issues, such as ED and hospital crowding.^14^

California operates under a 2-tiered EMS system that includes the state EMS Authority and 34 local EMS agencies (27 single-county and 7 multicounty), offering a unique model for studying APOT. While the role of the EMS Authority is to ensure equitable coordination, administration, and integration of the 34 local EMS agencies, each agency is responsible for delivering EMS within its own geographic region. In 2015, increasing strain on EMS systems across the state prompted the development of a standard methodology for APOT reporting, and reporting was mandated for all agencies beginning in 2019.^19,20^

Despite mandated reporting over the past 5 years, there are no published studies to date that examine the most recent patterns in APOT across California. A previous study from 2018 introduced the reporting metric APOT-1, which includes the 90th percentile ambulance offload time and number of offloads to a specific hospital.^21^ The study also examined offload times across 9 California local EMS agencies. However, it was published before APOT reporting was mandated by the state, and most EMS agencies were excluded. Another study from Los Angeles examined patterns in the total time ambulances were out of service from 2001 to 2002, but it was published more than 2 decades ago and evaluated 1 city only.^16^ The present study addresses these crucial gaps in the literature and offers a current statewide analysis of APOTs across all 34 local EMS agencies in California from January 1, 2021, through June 30, 2023. We hypothesized that there would be substantial variation in offload times between and within local EMS agencies, with the most severe offload delays showing little improvement over the study period.

## Methods

### Data Sources

This cohort study was approved by the University of California, San Francisco Institutional Review Board with a waiver of informed consent because the study did not constitute human participants research. The study followed the Strengthening the Reporting of Observational Studies in Epidemiology (STROBE) reporting guideline.

We used APOT reports from the California EMS Authority and local EMS agencies to examine patterns in APOT-1 and county-level population data from the US Census Bureau to determine mean ambulance offload volumes per 1000 population for each local EMS agency. Our primary source of APOT data was the EMS Authority website,^22^ which provides publicly available monthly, hospital-level APOT reports for each local EMS agency. EMS Authority data for April through June 2023 were aggregated at the local EMS agency level (rather than hospital level), and no reports were available for January through March 2023. To address these gaps in the data, we supplemented the EMS Authority reports with APOT reports directly from local EMS agencies. We contacted EMS medical directors and administrators in all 34 local EMS agencies to request hospital-level data for January through March 2023 (further details provided in the eMethods in Supplement 1). We included all entries that reported a 90th percentile offload time of 0, as a 0 time can be reported when the transfer of care from EMS to the ED occurs immediately upon arrival at the hospital. After merging data from the EMS Authority and local EMS agencies, missing data comprised 5% of monthly APOT reports. The final dataset included monthly offload volumes and APOT-1 90th percentile offload times by hospital (or by EMS agency for April through June 2023).

### Study Outcomes

Our primary outcomes, calculated for each local EMS agency, were (1) offload volumes, (2) mean annual offloads per 1000 population, (3) APOT-1 weighted mean accompanied by the standard deviation, and (4) APOT-1 median accompanied by the interquartile range. Offload volumes, weighted means (SDs), and medians (IQRs) were reported for the entire study period, annually, and biannually.

### Statistical Analysis

Given that our datasets were aggregated as monthly offload volumes and APOT-1 90th percentile offload times by hospital, all measures were calculated from these 90th percentile times. Using population data from the US Census Bureau, we calculated the mean annual offloads per 1000 population. We divided the total number of offloads for each local EMS agency over the entire study period by the product of the county (or counties for multicounty agencies) population size and the duration of observation and then multiplied the result by 1000. Mean annual offloads per 1000 population were normalized to account for missing months of data. This observation provides a standardized measure of how often offloads occur within a given population over a specific period and serves as an additional descriptor for each EMS agency.

Next, we examined APOT-1 weighted means. The APOT-1 weighted mean is the standard measure reported by the EMS Authority and accounts for variation in the number of offloads to each hospital. Weighted means for each hospital were calculated by multiplying the APOT-1 90th percentile time by the number of offloads to that hospital and then dividing the product by the total number of offloads for that local EMS agency. We then aggregated the weighted times by local EMS agency to derive the overall weighted mean for that agency. By multiplying the reported APOT-1 90th percentile time for a hospital by the percentage of offloads the hospital accounted for within its respective agency, we ensured that all offloads were weighted equally, revealing the severity of APOT in each local EMS agency as experienced by each offload or patient. In addition, and for completeness, we calculated the weighted standard deviation. To calculate the standard deviation, we first determined the weighted variance by finding the squared differences between each data point and the weighted mean, multiplying by their respective weights, and averaging these values. The standard deviation is the square root of the weighted variance. Of note, this method does not adjust for sample size, which may lead to a slight underestimation of the population variability.

Separately, we reported the median, or the central monthly APOT-1 time, across hospitals within a given local EMS agency, irrespective of the number of offloads. The median offers a measure of central tendency that is less influenced by outliers, providing insight into the typical offload time experienced by hospitals within each agency. When paired with the IQR, this measure provides insight into the range of APOT-1 times that most hospitals are experiencing within a given agency.

By incorporating these 2 distinct measures (weighted mean [SD] and median [IQR]), our analysis provides a comprehensive overview of offload times. The weighted mean offers an aggregate perspective by considering offload volumes, allowing us to discern overarching trends and compare APOT-1 times across local EMS agencies, while the median offers additional insight into the central tendency of offload times, mitigating the influence of outliers and providing a representative measure of typical offload times hospitals are experiencing within each agency. Together, these measures offer complementary perspectives, enabling a thorough understanding of offload times across California, from overall trends to typical experiences within the population served. All analyses were performed using Python, version 3.8.5 (Python Software Foundation). Modules used included Pandas, version 2.0.3; Seaborn, version 0.11.0; Matplotlib, version 3.3.2; and Geopandas, version 0.13.2.

## Results

To examine APOT-1 patterns across California from January 2021 through June 2023, we analyzed 5 913 399 offloads across 34 local EMS agencies. The APOT-1 weighted mean (SD) across the state was 42.8 (27.3) minutes, and the median monthly hospital-level APOT-1 was 28.9 minutes (IQR, 14.9-46.3 minutes).

Table 1 provides summary statistics by local EMS agency, including local EMS agency population, offload volumes, annual offloads per 1000 population, APOT-1 weighted means, and APOT-1 medians for all 34 local EMS agencies over the study period. Local EMS agency populations ranged from 54 641 residents in Tuolomne to 9 730 857 in Los Angeles. The highest and lowest offload volumes were reported in Los Angeles (1 172 724 offloads) and San Benito (5745 offloads), respectively. The highest annual ambulance offload rates were 94 offloads per 1000 population for the Tuolumne EMS agency, followed by Mountain Valley (92 offloads per 1000 population) and San Francisco (91 offloads per 1000 population). In contrast, the lowest annual offload rates were observed for San Diego (48 offloads per 1000 population), Imperial (45 offloads per 1000 population), and San Benito (34 offloads per 1000 population).

**Table 1.** Summary Statistics of Ambulance Offload Volumes and Times for the 34 California Local EMS Agencies, January 2021 to June 2023

Local EMS agency	Local EMS agency population, No.	Offload volume, No.	Mean annual offloads per 1000 population^a^	APOT-1, min		
				Weighted mean (SD)	Median (IQR)
Alameda	1 631 498	259 025	64	50.4 (7.7)	45.0 (34.0-56.8)
Central California	1 806 916	367 568	81	55.8 (5.4)	39.3 (27.6-51.1)
Coastal Valleys	573 145	104 772	73	21.4 (7.9)	10.0 (7.0-20.5)
Contra Costa	1 158 662	197 765	76	49.2 (0.4)	41.3 (31.6-55.0)
El Dorado^b^	192 902	25 105	65	16.4 (12.4)	12.6 (8.1-26.9)
Imperial^b^	179 045	15 441	45	26.4 (3.8)	22.2 (16.8-29.0)
Inland	2 227 204	395 543	71	51.4 (9.3)	23.1 (11.4-45.7)
Kern	914 427	161 936	71	54.4 (11.5)	48.0 (37.0-60.0)
Los Angeles	9 730 857	1 172 724	48	50.1 (31.1)	41.0 (27.7-56.0)
Marin	256 613	35 617	56	13.5 (1.2)	13.1 (10.1-14.3)
Merced	288 913	51 022	71	47.1 (10.8)	38.4 (24.5-52.5)
Monterey	433 588	57 229	59	17.5 (3.0)	15.6 (11.2-20.3)
Mountain Valley	106 187	87 878	92	34.0 (5.8)	27.6 (16.5-37.8)
Napa	134 617	22 732	75	15.2 (1.9)	14.4 (9.4-18.7)
North Coast	229 935	46 695	81	6.2 (2.3)	5.0 (3.0-7.9)
Northern California	77 435	12 384	71	7.5 (0.3)	6.0 (4.0-9.6)
Orange	3 148 913	450 010	57	32.3 (12.9)	30.7 (22.3-40.6)
Riverside	2 473 361	409 346	66	51.4 (17.6)	45.3 (27.9-69.4)
Sacramento	1 585 826	310 263	78	66.8 (16.8)	55.6 (33.8-74.2)
San Benito	67 509	5745	34	10.3 (1.4)	10.3 (7.0-12.7)
San Diego	3 273 860	393 684	48	46.3 (13.3)	43.4 (33.4-54.2)
San Francisco	809 566	185 057	91	36.2 (9.4)	30.0 (22.6-39.0)
San Joaquin	794 537	162 211	82	39.8 (6.4)	35.4 (30.8-40.0)
San Luis Obispo	281 050	35 697	56	13.3 (0.2)	12.9 (12.2-14.1)
San Mateo	731 477	94 520	52	14.6 (5.0)	12.4 (9.8-17.5)
Santa Barbara	440 898	68 249	62	13.5 (2.2)	11.8 (10.6-14.6)
Santa Clara^b^	1 880 367	186 248	50	27.6 (0.3)	20.2 (12.9-27.7)
Santa Cruz	262 087	34 596	59	20.6 (0.8)	15.9 (10.2-20.0)
Sierra–Sacramento Valley	1 251 155	274 463	88	23.9 (15.5)	11.0 (6.0-21.0)
Solano	449 585	82 698	74	23.3 (4.4)	23.2 (19.8-29.3)
Stanislaus^c^	551 793	41 157	75	41.6 (7.0)	38.3 (32.6-45.2)
Tuolumne	54 641	12 907	94	10.4 (1.4)	10.0 (8.1-10.0)
Ventura	833 977	115 966	56	22.8 (4.6)	20.6 (17.5-23.9)
Yolo	218 568	37 146	68	49.0 (21.6)	48.8 (30.1-70.4)
California total	39 051 114	5 913 399	60	42.8 (27.3)	28.9 (14.9-46.3)

Abbreviations: APOT-1, 90th percentile ambulance patient offload time; EMS, emergency medical services.

^a^ Mean annual offloads per 1000 population were determined using county population data from the US Census Bureau.

^b^ Local EMS agencies with offload results missing for more than 10% of the study period.

^c^ Stanislaus was part of the Mountain Valley local EMS agency until July 1, 2022. Results for the Stanislaus local EMS agency are reported from July 2022 through June 2023.

**Figure 1.**
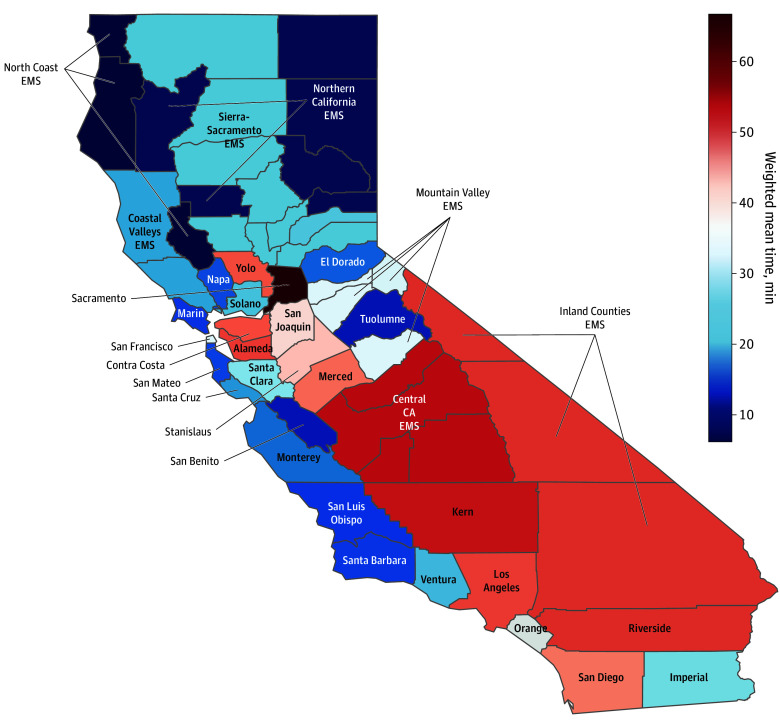
Heat Map of Ambulance Patient Offload Time Weighted Means for California’s 34 Local Emergency Medical Services (EMS) Agencies, January 2021 to June 2023

The APOT-1 weighted means also varied by local EMS agency. Figure 1 (with full results shown in Table 1) reveals the gradient of geographic variation in APOT-1 weighted means by agency. The highest APOT-1 weighted mean (SD) over the study period was 66.8 (16.8) minutes in Sacramento, while the lowest was 6.2 (2.3) minutes in the North Coast region. Of the total 34 local EMS agencies, 16 (47.1%, comprising 79.2% of all offloads) had an APOT-1 weighted mean greater than the 30-minute standard set by the state. Thirteen local EMS agencies (38.2%, comprising 69.0% of all offloads) experienced an APOT-1 weighted mean of more than 30 minutes for all 3 years of the study period, and 8 (24.2%) were among the top 10 APOT-1 weighted means for all 3 years. Of 33 local EMS agencies (we excluded Stanislaus as it was not an independent agency in 2021), 20 (60.6%) had an annual APOT-1 weighted mean that was worse in 2023 than in 2021.

**Figure 2.**
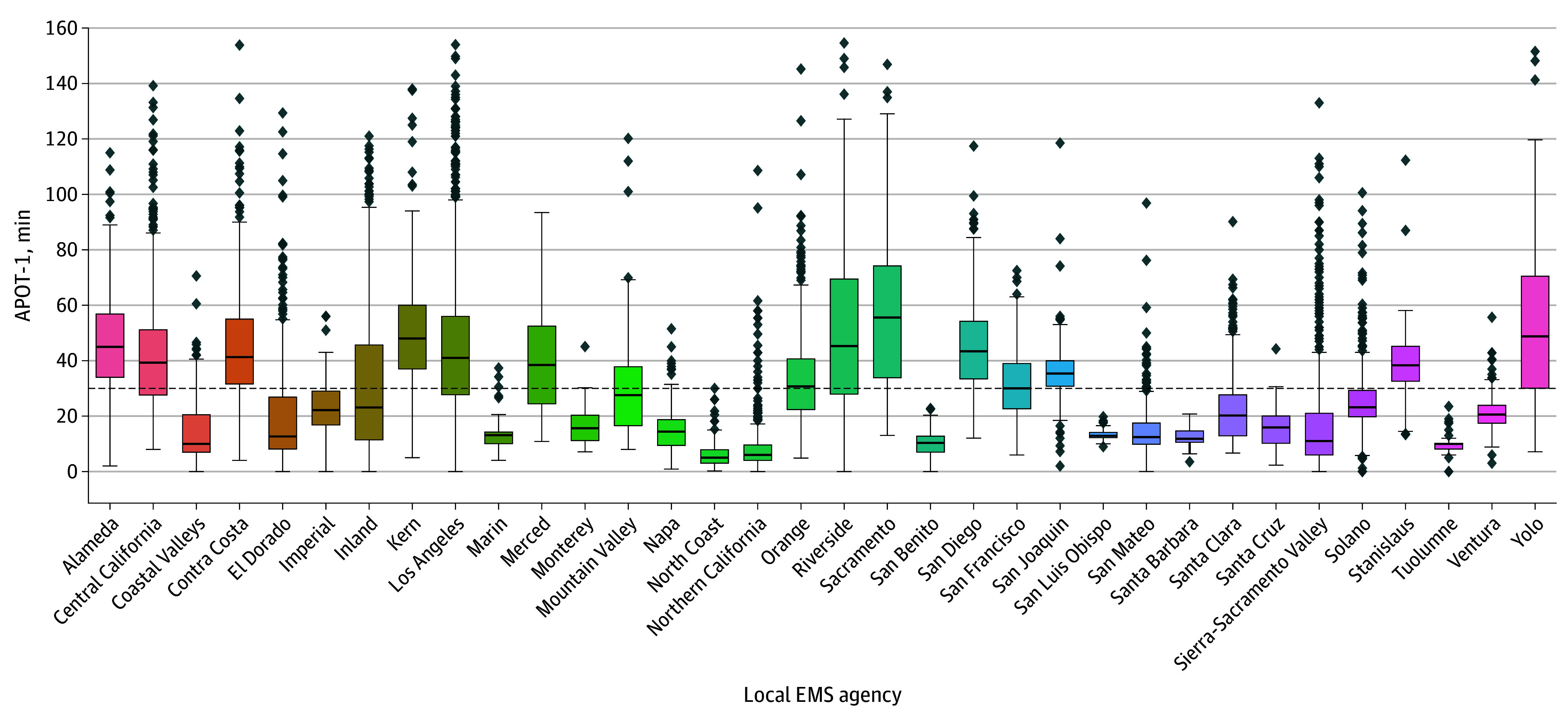
Variation in 90th Percentile Ambulance Patient Offload Time (APOT-1) by California Local Emergency Medical Services (EMS) Agency, January 2021 to June 2023 The horizontal bar inside the boxes indicates the median, and the lower and upper ends of the boxes are the first and third quartiles. The whiskers indicate values within 1.5 times the interquartile range from the upper or lower quartile, and data more extreme than the whiskers are plotted individually as outliers (diamonds).

Figure 2 (results provided in Table 1) depicts monthly hospital-level APOT-1 medians within local EMS agencies. Consistent with patterns in APOT-1 weighted means, the highest median APOT-1 time was 55.6 minutes (IQR, 33.8-74.2 minutes) in Sacramento, and the lowest was 5.0 minutes (IQR, 3.0-7.9 minutes) in the North Coast region. Variation, reported as IQR (Q1-Q3) within local EMS agencies, ranged from 41.5 minutes (27.9-69.4 minutes) for the Riverside local EMS agency to 1.9 minutes for both the San Luis Obispo (12.2-14.1 minutes) and Tuolumne (8.1-10.0 minutes) agencies. Overall, 13 of the 34 local EMS agencies (38.2%) had a median APOT-1 greater than the 30-minute standard.

Offload delays were also variable within local EMS agencies, with APOT-1 weighted means higher than corresponding medians for all 34 agencies (Table 1). For example, the weighted mean (SD) for Inland was 51.4 (9.3) minutes, while the median was 23.1 minutes (IQR, 11.4-45.7 minutes).

We further analyzed outcomes by year (Table 2, with biannual results shown in eTable 1 in Supplement 1). In 2021, the APOT-1 weighted mean (SD) in California was 41.8 (33.3) minutes; in 2022, 43.5 (23.3) minutes; and from January through June 2023, 43.1 (22.1) minutes. Annual results by local EMS agency were similar to overall patterns with a few notable exceptions. First, in 2021, the highest APOT-1 weighted mean (SD) was 73.4 (38.1) minutes for the Central California EMS agency (includes Fresno, Kings, Madera, and Tulare Counties), but this mean dropped to 45.0 (16.6) and 44.9 (5.4) minutes in 2022 and 2023, respectively. The APOT-1 weighted mean (SD) in Merced also dropped substantially over the study period from 55.1 (10.9) minutes in 2021 to 45.3 (9.6) minutes in 2022 and 33.0 (10.8) minutes in 2023. However, several other local EMS agencies experienced an increase in APOT-1 over the study period. In San Francisco, the APOT-1 weighted mean (SD) increased from 30.3 (7.2) minutes in 2021 to 44.8 (9.4) minutes in 2023 and in San Diego, from 38.7 (10.6) minutes in 2021 to 54.5 (13.3) minutes in 2023.

**Table 2.** Summary Statistics of Ambulance Offload Volumes and Times for the 34 California Local EMS Agencies by Year

Local EMS agency	2021			2022			2023 (January-June)			
	Offload volume, No.	APOT-1, min		Offload volume, No.	APOT-1, min		Offload volume, No.	APOT-1, min	
		Weighted mean (SD)	Median (IQR)		Weighted mean (SD)	Median (IQR)		Weighted mean (SD)	Median (IQR)
Alameda	100 345	53.2 (14.0)	47.2 (39.4-59.2)	107 024	50.3 (14.5)	43.8 (31.5-56.0)	51 656	45.5 (7.7)	43.0 (32.0-49.0)
Central California	139 645	73.4 (38.1)	43.8 (30.3-70.5)	151 035	45.0 (16.6)	37.3 (26.3-44.4)	76 888	44.9 (5.4)	40.5 (26.7-47.2)
Coastal Valleys	39 823	20.1 (9.0)	10.0 (7.0-22.0)	43 549	23.2 (12.9)	10.3 (7.0-20.5)	21 400	20.0 (7.9)	9.9 (7.0-18.3)
Contra Costa	78 749	44.7 (14.7)	39.6 (30.8-51.0)	99 501	52.8 (23.0)	41.8 (33.7-58.9)	19 515^a^	49.1 (0.4)	49.4 (49.0-49.4)
El Dorado	6073^a^	16.8 (18.0)	12.6 (7.8-27.2)	12 552	14.5 (13.8)	12.2 (8.0-24.6)	6480	19.6 (12.4)	17.3 (8.9-30.0)
Imperial	3287^a^	23.3 (8.2)	22.1 (20.6-25.8)	11 756	27.3 (7.6)	23.5 (17.2-29.8)	398^a^	25.4 (3.8)	12.9 (11.8-19.6)
Inland	153 026	55.6 (24.5)	19.7 (10.0-41.4)	160 138	49.6 (17.0)	31.0 (14.4-50.1)	82 379	47.2 (9.3)	22.2 (11.0-46.6)
Kern	63 296	53.4 (15.5)	49.0 (39.0-60.0)	63 961	54.6 (14.1)	46.0 (36.2-60.8)	34 679	55.7 (11.5)	52.4 (37.4-58.0)
Los Angeles	414 021	45.7 (55.3)	34.0 (22.0-47.9)	494 224	54.1 (22.5)	47.1 (34.0-63.0)	264 479	49.4 (31.1)	43.0 (30.0-55.0)
Marin	13 893	13.4 (1.2)	13.1 (11.7-14.0)	15 696	13.7 (1.5)	13.7 (10.0-14.9)	6028	13.1 (1.2)	12.6 (9.1-14.4)
Merced	20 124	55.1 (10.9)	48.8 (35.3-59.0)	22 137	45.3 (9.6)	35.6 (24.5-45.2)	8761	33.0 (10.8)	18.8 (15.8-25.9)
Monterey	24 610	16.7 (5.1)	14.2 (11.4-18.9)	19 929^a^	16.4 (3.6)	15.2 (10.1-19.0)	12 690	20.8 (3.0)	20.1 (11.7-21.7)
Mountain Valley	51 742	35.2 (8.4)	33.6 (28.0-40.7)	28 799	35.8 (12.7)	24.0 (12.9-38.4)	7337	18.7 (5.8)	19.0 (12.0-26.0)
Napa	9578	14.9 (4.8)	14.4 (9.6-18.4)	7798^a^	14.8 (3.7)	12.7 (9.2-18.6)	5356	16.3 (1.9)	15.8 (12.0-19.0)
North Coast	17 693	5.6 (2.9)	5.0 (2.8-6.9)	19 334	6.4 (2.8)	5.0 (4.0-7.6)	9668	7.0 (2.3)	6.0 (3.0-11.4)
Northern California	5897	7.3 (7.8)	6.0 (4.0-8.8)	5491	7.6 (8.9)	6.0 (4.0-10.6)	996^a^	7.5 (0.3)	7.3 (7.2-7.6)
Orange	163 308	29.8 (11.6)	28.0 (19.8-37.4)	186 657	34.5 (14.8)	33.3 (24.4-44.3)	100 045	32.3 (12.9)	32.1 (26.0-46.5)
Riverside	158 323	52.1 (26.9)	45.1 (32.8-72.0)	166 719	52.6 (31.1)	44.7 (22.2-67.8)	84 304	47.8 (17.6)	49.7 (30.1-65.9)
Sacramento	124 590	62.0 (22.8)	49.0 (32.8-69.1)	125 941	69.2 (26.0)	56.6 (33.6-78.9)	59 732	71.6 (16.8)	69.6 (55.0-80.0)
San Benito	2220	9.3 (3.2)	10.6 (6.6-12.6)	2439	10.7 (1.8)	11.0 (8.4-16.8)	1086	11.3 (1.4)	8.6 (5.8-11.8)
San Diego	141 296	38.7 (10.6)	37.8 (29.7-46.5)	160 577	48.2 (14.6)	45.7 (35.2-57.9)	91 811	54.5 (13.3)	52.8 (43.2-63.7)
San Francisco	73 056	30.3 (7.2)	26.0 (21.0-33.0)	72 967	37.7 (10.4)	32.0 (24.0-40.0)	39 034	44.8 (9.4)	44.3 (32.2-53.0)
San Joaquin	64 355	38.1 (6.4)	35.0 (30.7-38.9)	67 767	39.9 (7.3)	35.6 (29.5-40.2)	30 089	43.2 (6.4)	38.0 (33.8-44.6)
San Luis Obispo	14 978	13.4 (1.4)	13.2 (12.4-13.8)	16 293	13.5 (1.9)	12.9 (12.0-14.4)	4426^a^	12.3 (0.2)	12.4 (12.2-12.4)
San Mateo	34 498	11.8 (3.0)	10.0 (9.0-14.4)	38 682	15.1 (4.1)	13.6 (10.5-18.5)	21 340	18.4 (5.0)	14.9 (11.2-23.4)
Santa Barbara	26 308	14.1 (2.7)	12.8 (10.9-14.7)	29 007	13.3 (2.9)	11.4 (10.2-15.1)	12 934	12.9 (2.2)	10.8 (9.6-13.0)
Santa Clara	64 325^a^	24.2 (12.6)	17.7 (12.6-23.2)	98 567	29.9 (16.3)	21.0 (13.5-30.2)	23 356^a^	27.3 (0.3)	27.3 (27.1-27.5)
Santa Cruz	15 933	21.2 (3.1)	15.4 (10.3-20.9)	14 942	20.4 (2.9)	15.7 (10.1-19.0)	3721^a^	19.0 (0.8)	19.5 (18.7-19.6)
Sierra–Sacramento	109 411	21.7 (17.5)	10.0 (6.0-17.2)	113 873	24.4 (19.1)	11.0 (7.0-23.0)	51 179	27.7 (15.5)	11.0 (6.0-28.1)
Solano	34 425	22.4 (4.4)	22.4 (18.3-26.1)	34 744	22.7 (4.8)	23.3 (20.0-30.1)	13 529	27.1 (4.4)	29.4 (26.7-34.0)
Stanislaus^b^	NA	NA	NA	26 902	41.9 (7.0)	38.3 (27.1-44.9)	14 255^a^	41.1 (7.0)	40.6 (34.2-45.7)
Tuolumne	5299	11.7 (5.9)	8.5 (7.0-11.9)	5108	8.9 (2.7)	10.0 (9.0-10.0)	2500	10.7 (1.4)	10.0 (10.0-11.5)
Ventura	44 650	21.6 (4.6)	20.1 (17.5-23.0)	48 400	23.8 (4.9)	21.7 (18.1-24.8)	22 916	22.9 (4.6)	19.9 (15.7-24.8)
Yolo	14 411	46.9 (24.9)	48.3 (28.8-69.0)	15 001	52.4 (28.7)	49.0 (32.6-69.6)	7734	46.3 (21.6)	41.5 (30.7-78.8)
California total	2 233 188	41.8 (33.3)	26.0 (13.9-43.0)	2 487 510	43.5 (23.3)	30.5 (15.6-49.0)	1 192 701	43.1 (22.1)	32.0 (16.1-49.4)

Abbreviations: APOT-1, 90th percentile ambulance patient offload time; EMS, emergency medical services; NA, not available.

^a^ Local EMS agencies with offload results missing for 1 or more months.

^b^ Stanislaus was part of the Mountain Valley local EMS agency until July 1, 2022. Results for the Stanislaus local EMS agency are reported from July 2022 through June 2023.

**Figure 3.**
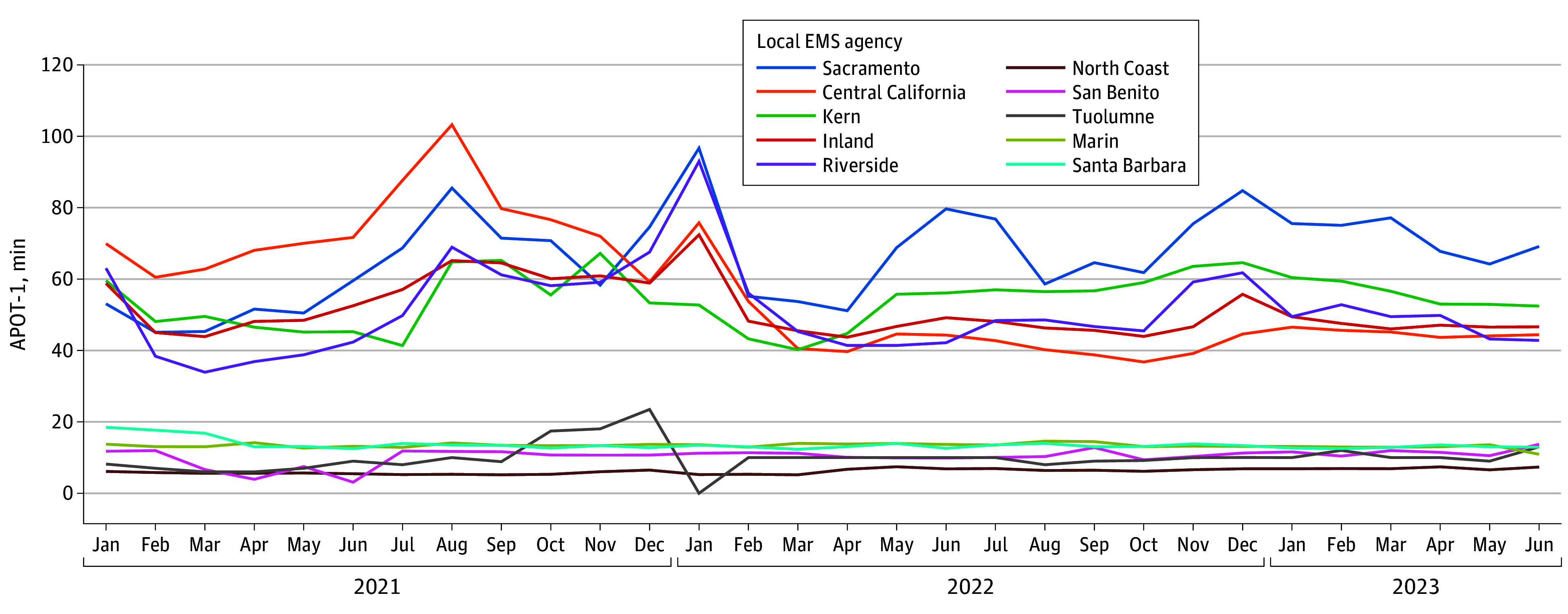
Patterns in Monthly 90th Percentile Ambulance Patient Offload Time (APOT-1) Weighted Means for the 5 California Local Emergency Medical Services (EMS) Agencies With the Longest and Shortest Times, January 2021 to June 2023

Figure 3 displays temporal patterns in monthly APOT-1 weighted means for the 5 local EMS agencies with the highest and lowest weighted means over the study period. Only agencies that reported for all 30 months of the study period were included, with complete monthly results by agency shown in eTable 2 in Supplement 1. For the 5 local EMS agencies with the highest APOT-1 weighted means, fluctuations in monthly means were relatively uniform. Notable spikes in APOT-1 were observed in August 2021, January 2022, and December 2022. The APOT-1 weighted means were more volatile among the 5 local EMS agencies with the highest means compared with the lowest. For example, monthly means (SDs) in Central California (1 of the 5 highest) ranged from 36.8 (7.2) minutes in October 2022 to 103.2 (62.7) minutes in August 2021. In contrast, monthly means (SDs) in Marin (1 of the 5 lowest) ranged from 14.6 (0.9) minutes in August 2022 to 10.9 (0.0) minutes in June 2023.

## Discussion

In this cohort study, we provide the first, to our knowledge, comprehensive and up-to-date illustration of the widespread variation in ambulance offload delays across California. Nearly one-half of all local EMS agencies (16 of 34, representing 79% of all offloads) had an APOT-1 weighted mean greater than the 30-minute standard set by the state. Similarly, 13 of 34 EMS agencies had a median APOT-1 that did not meet the state standard; in other words, at least 50% of monthly hospital offload reports for those agencies were longer than 30 minutes.

We found that long offload times were more common among certain regions and hospitals. Notably, 13 of the 34 local EMS agencies (comprising 69% of all offloads) experienced an APOT-1 weighted mean of more than 30 minutes for all 3 years of the study period and 8 were among the top 10 APOT-1 weighted means across all 3 years, suggesting that the most severe regional offload delays are not random but are consistently more severe in certain geographic regions. Moreover, long offload times have persisted, and potentially worsened, even as the COVID-19 pandemic subsided in 2022 and 2023. We found that 20 local EMS agencies had an annual APOT-1 weighted mean that was worse in 2023 than in 2021. These findings are particularly sobering as they reveal that pandemic-related surges in ED crowding and boarding^6,23^ have not fully recovered to prepandemic levels and may persist without rigorous system-level intervention.

Offload delays were also highly variable within local EMS agencies, and APOT-1 weighted means were higher than corresponding medians for all 34 agencies. Notably, the weighted mean for the Inland agency was 51.4 minutes, while the median was just 23.1 minutes (less than one-half of the weighted mean). Given that the weighted mean accounts for each offload equally while the median represents the middle monthly APOT-1 time by hospital, the considerable 28.3-minute difference between the 2 may have been driven by a small proportion of monthly reports with high offload volumes and long offload times. Patient characteristics and disposition may also have a significant impact on APOT. For instance, EMS cases deemed “alerts” from the field, such as patients with ST-segment elevation myocardial infarction, stroke, and trauma, will likely not experience APOT delays. Further examination of hospital, seasonal, and patient characteristics within agencies may offer valuable guidance to EMS and hospitals as they develop targeted strategies to reduce APOT within their jurisdictions. For instance, agencies experiencing high APOT-1 weighted means and medians may require broad, regional, or local interventions, while those with high weighted means but much lower medians (eg, Inland) may direct efforts toward specific hospitals or improve planning for seasonal or other unexpected surges.

Finally, our temporal analysis showed that monthly fluctuations in offload times were relatively consistent across local EMS agencies. However, it is important to note that our study was conducted during the COVID-19 pandemic, and some of these monthly patterns may have been influenced by surges in COVID-19 infections (ie, the spike in APOT-1 in August 2021 may be due to increased infections during the Delta wave and in January 2022 due to the Omicron wave). Despite difficulty disentangling pandemic-related increases in APOT delays from other underlying factors, national data suggest that EMS activations were increasing prior to the COVID-19 pandemic.^24^ Additionally, 90% of EDs regularly reported overcrowding even before the COVID-19 pandemic, with minimal capacity to absorb spikes in demand,^7,25^ suggesting that an overall increase in offload delays may have persisted regardless of the pandemic.

While this is the first study to examine longitudinal patterns in APOT across California, it is not the first to suggest that hospital and ED crowding have become increasingly concerning in recent years.^26,27^ Our findings confirm that offload delays in California have, in fact, shown little to no improvement, even as the COVID-19 pandemic receded in 2022 and 2023. In fact, from April through September 2023, the California EMS Authority reported more than 65 000 hours in delays greater than 30 minutes.^28^ Despite differences in EMS systems across the country, we would expect the overall implications of our findings to extend nationally. Issues with APOT, ED crowding, and boarding are not confined to California.^29,30,31,32^ By investigating the severity and variation in APOT across California, we have gained a better understanding of potential drivers and, eventually, solutions to improve timely access to emergency care and encourage conversation and collaboration among stakeholders nationally.

Successful and sustainable interventions aimed at improving ambulance offload delays and ED crowding are 2-fold. Short-term and stopgap solutions to mitigate offload delays may include appointing EMS personnel as patient flow coordinators or liaisons in the ED^33,34,35,36^ or hiring an offload nurse to ensure the efficient transfer of care from EMS to the ED team.^37^ Such interventions have already been implemented in individual hospitals. For example, in April 2022, Community Regional Medical Center in Fresno implemented an offload zone, or ED hallway with additional beds staffed by licensed vocational nurses, to promote the efficient transfer of care from EMS to the ED team. Similarly, Santa Clara County’s Valley Medical Center launched the Ambulance Patient Offload Delay Patient Triage Pilot Program through which an ambulance nurse assigns incoming 911 ambulance patients a severity score to determine which patients should be assigned directly to ED beds vs sent to the waiting room.

While these targeted interventions may help alleviate the immediate burden of offload delays in the short term, sustainable long-term relief will require comprehensive, system-level initiatives that tackle the root causes of ED crowding (eg, inpatient crowding, discharge delays, staffing shortages, hospital throughput).^38^ At its core, long APOTs reflect downstream issues associated with hospital crowding and are a symptom, rather than the root, of the problem.

### Limitations

Our study has several limitations. First, the majority of offload data used in this study was obtained from publicly available APOT reports from the California EMS Authority; however, there were sporadic gaps in the data, and no EMS Authority reports were available for January through March 2023 (the period during which the EMS Authority took over APOT reporting in the California EMS Information System). We addressed these gaps by obtaining monthly hospital-level APOT reports directly from local EMS agencies, and there may be variation between the 2 sources for which we were unable to account. Second, EMS Authority data between April and June 2023 are at the local EMS agency rather than the hospital level. These agency-level reports do not account for hospital-level variation, and APOT-1 monthly means for individual hospitals during this 3-month period are not included in overall medians and interquartile ranges. However, the goal of this study was to provide an overview of APOT across the state, and we do not expect that these missing data played a substantial role in the overall metrics. Third, EMS Authority data from April through June 2023 only include records submitted using the National EMS Information System, version 3.4 standard. Some EMS agencies transitioned to National EMS Information System, version 3.5 in early 2023 and have decreased representation during these months. Fourth, 100% of the Los Angeles EMS agency’s offload data and approximately 60% of San Diego’s data were missing from the 2023 EMS Authority and California EMS Information System reports and instead were obtained directly from the respective local EMS agencies. Fifth, data for the latter months of 2023 were not yet available from the EMS Authority, limiting our 2023 findings to January through June. Given that this study was intended to be a general overview of APOT in California and our biannual results (eTable 1 in Supplement 1) do not show any remarkable differences between first and second half data in 2021 and 2022, we do not expect the results for July to December 2023 to substantially change our overall findings. Finally, we examined annual offloads per 1000 population by local EMS agency using county populations, and agencies may transport patients outside of their county or region. With this said, only a small percentage of all transports in California are to hospitals outside of an agency’s jurisdiction, so we do not expect this to substantially affect our results.

## Conclusions

In this cohort study, we found significant variation in the severity of ambulance offload delays between and within California local EMS agencies, with approximately one-half of agencies consistently reporting offload times greater than the 30-minute state standard. The most severe delays are persistent in certain geographic regions, reflecting greater ED and hospital crowding that has not improved in recent years. Our findings should spur collaborative efforts among stakeholders to address the deeper issues causing ambulance offload delays across the state.

## Notes

Supplement 1. **eMethods.** Additional Details on Methods for Data Collection **eTable 1.** Biannual Summary Statistics of Ambulance Offload Volumes and Times **eTable 2.** Monthly APOT-1 Weighted Means for the 5 Local EMS Agencies With the Highest and Lowest APOT-1 Weighted Means Over the Study Period

Supplement 2.
Data Sharing Statement
