# Error in Results and Discussion

**DOI:** 10.1001/jamanetworkopen.2024.59486

**Published:** 2025-01-14

In the Original Investigation titled “Patterns in California Ambulance Patient Offload Times by Local Emergency Medical Services Agency,”^1^ published December 16, 2024, the Results and Discussion incorrectly stated that among 13 emergency medical services agencies with an APOT-1 weighted mean of more than 30 minutes for all 3 years of the study period, 7 were among the top 10 APOT-1 weighted means for all 3 years; in reality, 8 of the 13 were among the top 10 for all 3 years. This article has been corrected.^1^

## References

[1] Feldmeier M, Reyes KP, Chen C, . Patterns in California ambulance patient offload times by local emergency medical services agency. JAMA Netw Open. 2024;7(12):e2451022. doi:10.1001/jamanetworkopen.2024.51022 39680409 PMC11650417

