# Supplementary material for: Patterns in California Ambulance Patient Offload Times by Local Emergency Medical Services Agency
Source: JAMA Netw Open. 2024 Dec 16;7(12):e2451022. doi: 10.1001/jamanetworkopen.2024.51022 (PMC11650417; doi:10.1001/jamanetworkopen.2024.51022)
Supplement: Supplement 1. — eMethods. Additional Details on Methods for Data Collection eTable 1. Biannual Summary Statistics of Ambulance Offload Volumes and Times eTable 2. Monthly APOT-1 Weighted Means for the 5 Local EMS Agencies With the Highest and Lowest APOT-1 Weighted Means Over the Study Period [file jamanetwopen-e2451022-s001.pdf]

Supplemental Online Content

Feldmeier M, Reyes KP, Chen C, et al. Patterns in California ambulance patient offload times by local emergency medical services agency. *JAMA Netw Open*. 2024;7(12):e2451022. doi:10.1001/jamanetworkopen.2024.51022

eMethods. Additional Details on Methods for Data Collection

eTable 1. Biannual Summary Statistics of Ambulance Offload Volumes and Times

eTable 2. Monthly APOT-1 Weighted Means for the 5 Local EMS Agencies With the Highest and Lowest APOT-1 Weighted Means Over the Study Period

This supplemental material has been provided by the authors to give readers additional information about their work.

eMethods. Additional Details on Methods for Data Collection

We sought to collect hospital-level data on ambulance patient offload times (APOT) from local emergency medical services (EMS) agencies for January through March 2023 as this was not available through EMSA during this period. Data collection occurred from February 1, 2024, to April 17, 2024. The steps were as follows:

1. From February to March 2024, we sought to elicit initial information on APOT reporting practices by local EMS agency. We conducted internet searches of all public-facing local EMS agency websites and searched for mentions of APOT data reporting using key words such as "ambulance patient offload times," "APOT," "ambulance offload delay," and "EMS reports." We also reviewed previous APOT data reports and press releases. We found that the availability and presentation of public-facing APOT data varied significantly by local EMS agency— from no mention of APOT to weekly reports and interactive APOT dashboards. We collected contact information for 2-3 individuals in each local EMS agency who could provide APOT data, namely EMS administrators, medical directors, data analysts, and statisticians. If email contacts were unavailable, we called the general local EMS agency contact to request further information.
    a. From local EMS agency websites, we downloaded any publicly available datasets that included monthly hospital-level 90th percentile APOT times and offload volumes for January to March 2023 (5 of 34 local EMS agencies).
2. Next, we contacted all 34 local EMS agencies to request hospital-level data for January through March 2023. Through email correspondences, we gathered this data for 28/34 local EMS agencies.

© 2024 Feldmeier et al. *JAMA Network Open*.

3. Given that datasets were sent in various document formats (PDFs, CSVs, Excel sheets) as well as time-formatting for APOT times, we proceeded to standardize all local EMS agency data collected into an Excel format that could be merged with the EMSA data.

© 2024 Feldmeier et al. *JAMA Network Open*.

eTable 1. Biannual Summary Statistics of Ambulance Offload Volumes and Times

1a. Biannual Summary Statistics of Ambulance Offload Volumes and Times, 2021

	January to June						July to December					
**Local EMS Agency**	**Offloads**	**Weighted Mean (SD)**	**Median**	**Q1**	**Q3**	**IQR**	**Offloads**	**Weighted Mean (SD)**	**Median**	**Q1**	**Q3**	**IQR**
**Alameda**	46,768	51.8 (13.9)	51.8	46.3	40.0	56.2	53,577	54.4 (14.0)	49.3	37.2	61.8	24.6
**Central California**	74,603	67.7 (26.9)	67.7	40.9	29.8	65.8	65,042	79.9 (47.0)	45.3	31.0	81.8	50.8
**Coastal Valleys**	19,154	17.5 (7.6)	17.5	10.3	6.2	18.4	20,669	22.6 (9.5)	10.0	7.0	25.5	18.5
**Contra Costa**	37,058	42.7 (12.3)	42.7	37.3	29.1	49.1	41,691	46.4 (16.3)	41.8	33.2	53.1	20.0
**El Dorado**	--	--	--	--	--	--	6,073	16.8 (18.0)	12.6	7.8	27.2	18.9
**Imperial**	--	--	--	--	--	--	3,287[a]	23.3 (8.2)	22.1	20.6	25.8	5.1
**Inland**	73,343	49.6 (20.5)	49.6	17.9	9.4	36.0	79,683	61.1 (26.4)	31.7	15.6	63.8	49.0
**Kern**	30,623	48.9 (10.2)	48.9	42.0	35.0	52.2	32,673	57.6 (18.2)	55.0	46.5	68.2	21.7
**Los Angeles**	173,207	39.3 (17.4)	39.4	32.0	21.0	44.0	240,814	50.3 (70.7)	36.0	23.0	50.5	28.0
**Marin**	6,630	13.3 (1.4)	13.3	13.0	11.2	13.9	7,263	13.5 (1.1)	13.4	12.1	14.0	1.9
**Merced**	9,035	54.5 (9.5)	54.5	52.5	34.0	60.9	11,089	55.6 (11.9)	45.1	37.4	55.6	18.2
**Monterey**	11,809	16.7 (5.1)	16.7	14.2	12.0	19.3	12,801	16.8 (5.0)	13.9	11.0	18.9	7.9
**Mountain Valley**	24,775	32.1 (5.8)	32.1	31.1	26.7	35.7	26,967	38.0 (9.4)	34.5	28.7	43.6	14.8
**Napa**	4,471	14.0 (4.1)	14.0	12.8	8.3	18.0	5,107	15.6 (5.3)	15.0	10.5	20.5	10.4
**North Coast**	8,299	5.7 (2.6)	5.7	5.0	2.2	6.6	9,394	5.6 (3.1)	4.0	3.0	7.3	4.3
**Northern California**	2,655	6.5 (4.4)	6.5	5.4	4.2	8.5	3,242	8.0 (9.7)	6.0	4.0	9.2	6.0
**Orange**	72,324	26.8 (10.8)	26.8	23.7	18.2	32.3	90,984	32.1 (11.7)	30.6	22.8	39.7	16.9
**Riverside**	75,729	42.5 (21.3)	42.5	38.7	29.5	49.0	82,594	60.8 (28.4)	64.3	37.7	82.5	44.7
**Sacramento**	59,241	51.1 (16.0)	51.1	43.0	28.4	57.0	65,349	71.8 (23.5)	61.8	36.6	84.9	48.3
**San Benito**	1,078	7.3 (3.5)	7.3	5.1	3.2	8.0	1,142	11.2 (1.1)	11.4	10.6	12.7	4.1
**San Diego**	58,411	34.3 (9.0)	34.3	34.0	27.4	39.8	82,885	41.8 (10.5)	41.1	33.2	49.8	16.6
**San Francisco**	34,577	27.9 (5.7)	27.9	24.0	20.6	30.0	38,479	32.4 (7.8)	29.0	22.0	37.0	15.0
**San Joaquin**	30,658	35.5 (5.7)	35.5	32.6	29.3	36.5	33,697	40.4 (6.2)	37.7	33.9	41.3	7.4
**San Luis Obispo**	7,022	13.0 (1.0)	13.0	12.8	12.4	14.0	7,956	13.7 (1.7)	13.3	12.4	13.7	1.3
**San Mateo**	16,059	11.5 (2.9)	11.5	10.0	9.0	14.3	18,439	12.1 (3.1)	10.2	9.4	14.8	7.0
**Santa Barbara**	12,155	15.2 (3.1)	15.2	14.3	11.5	16.1	14,153	13.3 (1.9)	12.1	10.7	13.5	2.8
**Santa Clara**	41,339	24.3 (13.6)	24.3	17.2	12.6	22.5	22,986[a]	24.1 (10.6)	18.6	12.6	25.9	13.2
**Santa Cruz**	8,640	20.9 (3.3)	20.9	16.8	10.4	21.9	7,293	21.6 (2.7)	15.4	10.4	19.3	10.2
**Sierra-Sacramento**	51,979	17.6 (11.1)	17.6	9.0	6.0	16.0	57,432	25.3 (21.1)	10.5	6.0	20.0	14.0
**Solano**	16,889	21.6 (3.9)	21.6	21.7	18.0	25.2	17,536	23.2 (4.7)	23.0	19.0	27.0	8.2
**Stanislaus[b]**	--	--	--	--	--	--	--	--	--	--	--	--
**Tuolumne**	2,236	7.2 (1.1)	7.0	6.2	7.9	1.6	3,063	15.0 (5.8)	13.7	9.1	17.9	8.8
**Ventura**	21,335	20.4 (3.8)	19.2	16.3	21.9	5.5	23,315	22.7 (4.9)	21.2	18.2	23.5	5.3
**Yolo**	6,958	41.7 (21.5)	41.4	25.5	62.4	36.9	7,453	51.8 (26.8)	57.1	33.7	80.8	47.1

Note: APOT-1 indicates 90th percentile offload time. EMS indicates emergency medical services. APOT-1 weighted mean, median, Q1, Q3, and IQR in minutes.

[a] Local EMS agency with offload results missing for one or more months.

[b] Stanislaus was part of the Mountain Valley local EMS agency until July 1, 2022. Results for the Stanislaus local EMS agency are reported from July 2022 through June 2023.

© 2024 Feldmeier et al. *JAMA Network Open*.

1b. Biannual Summary Statistics of Ambulance Offload Volumes and Times, 2022

	January to June						July to December					
**Local EMS Agency**	**Offloads**	**Weighted Mean (SD)**	**Median**	**Q1**	**Q3**	**IQR**	**Offloads**	**Weighted Mean (SD)**	**Median**	**Q1**	**Q3**	**IQR**
**Alameda**	53,286	49.1 (15.7)	44.8	35.0	56.0	21.0	53,738	51.4 (13.1)	42.0	28.4	55.0	26.6
**Central California**	73,182	49.8 (21.2)	38.8	28.4	50.4	22.0	77,853	40.4 (8.3)	35.1	26.1	43.3	17.2
**Coastal Valleys**	20,946	22.9 (13.2)	10.1	6.2	20.8	14.6	22,603	23.6 (12.5)	10.3	7.0	20.0	13.0
**Contra Costa**	55,412	53.1 (24.6)	39.4	31.4	56.2	24.8	44,089	52.3 (20.9)	45.0	36.5	65.0	28.6
**El Dorado**	5,922	14.0 (14.9)	11.4	7.9	22.5	14.6	6,630	15.0 (12.7)	14.2	8.1	29.2	21.1
**Imperial**	6,632	24.8 (7.3)	20.6	9.9	24.6	14.7	5,124	30.4 (6.6)	26.0	20.5	33.0	12.5
**Inland**	76,816	51.5 (19.0)	38.2	16.4	55.4	38.9	83,322	47.8 (14.7)	28.0	13.4	46.7	33.3
**Kern**	30,512	49.1 (13.1)	42.0	34.0	52.0	18.0	33,449	59.7 (13.2)	53.5	40.0	64.0	24.0
**Los Angeles**	240,484	50.7 (21.4)	44.2	31.2	58.0	26.8	253,740	57.3 (23.0)	50.3	36.4	67.0	30.6
**Marin**	7,594	13.7 (1.4)	13.7	12.2	14.6	2.4	8,102	13.7 (1.6)	13.4	10.0	14.9	4.9
**Merced**	10,893	45.0 (11.2)	35.9	25.9	44.9	19.0	11,244	45.7 (7.7)	33.4	24.3	45.4	21.1
**Monterey**	6,551[a]	15.4 (3.4)	14.5	10.0	17.5	7.5	13,378	16.9 (3.5)	15.4	11.9	19.5	7.6
**Mountain Valley**	24,778	39.4 (9.2)	38.3	31.0	44.8	13.8	4,021	13.1 (6.9)	14.1	11.0	20.5	9.5
**Napa**	2,521[a]	14.1 (4.3)	9.6	8.0	15.4	7.4	5,277	15.2 (3.3)	14.0	9.9	19.1	9.2
**North Coast**	9,316	6.1 (3.1)	4.5	3.0	7.6	4.6	10,018	6.6 (2.4)	5.6	4.0	7.6	3.6
**Northern California**	2,579	7.1 (4.9)	6.3	4.0	10.8	6.8	2,912	8.0 (11.3)	6.0	4.0	10.0	5.9
**Orange**	89,686	33.1 (14.0)	30.4	22.4	40.5	18.1	96,971	35.8 (15.5)	36.2	27.4	47.3	19.9
**Riverside**	79,832	53.4 (34.1)	47.1	30.2	66.3	36.1	86,887	51.9 (28.1)	43.8	20.0	68.3	48.3
**Sacramento**	62,746	68.1 (28.8)	54.3	35.0	72.4	37.4	63,195	70.3 (22.9)	57.7	30.2	79.2	49.0
**San Benito**	1,184	10.6 (1.5)	10.0	7.4	16.8	9.3	1,255	10.7 (2.0)	11.4	9.3	17.5	8.2
**San Diego**	69,470	44.8 (14.4)	43.2	32.7	51.9	19.2	91,107	50.8 (14.2)	52.1	40.0	62.6	22.6
**San Francisco**	37,844	35.3 (9.3)	31.0	24.0	38.8	14.8	35,123	40.2 (10.8)	33.0	25.5	43.5	18.0
**San Joaquin**	33,587	38.5 (6.8)	34.5	30.5	38.0	7.5	34,180	41.3 (7.5)	35.9	28.5	42.4	13.8
**San Luis Obispo**	7,835	14.2 (1.8)	14.1	12.4	15.0	2.6	8,458	12.9 (1.8)	12.5	11.7	13.3	1.6
**San Mateo**	18,259	14.9 (4.1)	13.6	10.8	17.3	6.5	20,423	15.2 (4.1)	13.7	10.3	18.8	8.5
**Santa Barbara**	14,298	13.1 (3.0)	11.1	10.0	13.8	3.7	14,709	13.5 (2.9)	11.9	10.4	15.8	5.3
**Santa Clara**	47,670	29.9 (16.8)	20.7	13.5	30.3	16.9	50,897	29.8 (15.9)	21.2	13.5	28.8	15.3
**Santa Cruz**	7,228	21.1 (3.0)	15.9	9.9	20.1	10.2	7,714	19.7 (2.6)	15.0	10.9	18.5	7.6
**Sierra-Sacramento**	55,774	23.1 (17.7)	10.0	6.2	20.0	13.8	58,099	25.6 (20.3)	11.0	7.0	25.5	18.5
**Solano**	15,259	22.4 (4.6)	23.0	19.9	30.1	10.2	19,485	22.9 (5.0)	23.3	20.5	30.1	9.6
**Stanislaus[b]**	--	--	--	--	--	--	26,902	41.9 (9.4)	38.3	27.1	44.9	17.8
**Tuolumne**	2,456	8.3 (3.7)	10.0	10.0	10.0	0.0	2,652	9.4 (0.9)	10.0	8.5	11.0	2.5
**Ventura**	23,343	23.9 (5.1)	22.0	19.1	24.8	5.7	25,057	23.8 (4.7)	20.9	17.6	24.6	7.0
**Yolo**	7,348	55.0 (33.3)	49.0	35.2	69.7	34.4	7,653	49.9 (23.0)	47.8	29.3	69.6	40.3

Note: APOT-1 indicates 90th percentile offload time. EMS indicates emergency medical services. APOT-1 weighted mean, median, Q1, Q3, and IQR in minutes.

[a] Local EMS agencies with offload results missing for one or more months.

[b] Stanislaus was part of the Mountain Valley local EMS agency until July 1, 2022. Results for the Stanislaus local EMS agency are reported from July 2022 through June 2023.

© 2024 Feldmeier et al. *JAMA Network Open*.

1c. Biannual Summary Statistics of Ambulance Offload Volumes and Times, 2023

	January to June					
**Local EMS Agency**	**Offloads**	**Weighted Mean (SD)**	**Median**	**Q1**	**Q3**	**IQR**
**Alameda**	51,656	45.5 (7.7)	43.0	32.0	49.0	17.0
**Central California**	76,888	44.9 (5.4)	40.5	26.7	47.2	20.5
**Coastal Valleys**	21,400	20.0 (7.9)	9.9	7.0	18.3	11.3
**Contra Costa**[a]	19,515	49.1 (0.4)	49.4	49.0	49.4	0.4
**El Dorado**	6,480	19.6 (12.4)	17.3	8.9	30.0	21.1
**Imperial**[a]	398	25.4 (3.8)	12.9	11.8	19.6	7.9
**Inland**	82,379	47.2 (9.3)	22.2	11.0	46.6	35.6
**Kern**	34,679	55.7 (11.5)	52.4	37.4	58.0	20.6
**Los Angeles**	264,479	49.4 (31.1)	43.0	30.0	55.0	25.0
**Marin**	6,028	13.1 (1.2)	12.6	9.1	14.4	5.3
**Merced**	8,761	33.0 (10.8)	18.8	15.8	25.9	10.2
**Monterey**	12,690	20.8 (3.0)	20.1	11.7	21.7	10.0
**Mountain Valley**	7,337	18.7 (5.8)	19.0	12.0	26.0	14.0
**Napa**	5,356	16.3 (1.9)	15.8	12.0	19.0	7.0
**North Coast**	9,668	7.0 (2.3)	6.0	3.0	11.4	8.4
**Northern California**[a]	996	7.5 (0.3)	7.3	7.2	7.6	0.4
**Orange**	100,045	32.3 (12.9)	32.1	26.0	46.5	20.5
**Riverside**	84,304	47.8 (17.6)	49.7	30.1	65.9	35.8
**Sacramento**	59,732	71.6 (16.8)	69.6	55.0	80.0	25.0
**San Benito**	1,086	11.3 (1.4)	8.6	5.8	11.8	6.0
**San Diego**	91,811	54.5 (13.3)	52.8	43.2	63.7	20.5
**San Francisco**	39,034	44.8 (9.4)	44.3	32.2	53.0	20.8
**San Joaquin**	30,089	43.2 (6.4)	38.0	33.8	44.6	10.8
**San Luis Obispo**[a]	4,426	12.3 (0.2)	12.4	12.2	12.4	0.3
**San Mateo**	21,340	18.4 (5.0)	14.9	11.2	23.4	12.2
**Santa Barbara**	12,934	12.9 (2.2)	10.8	9.6	13.0	3.4
**Santa Clara**[a]	23,356	27.3 (0.3)	27.3	27.1	27.5	0.4
**Santa Cruz**[a]	3,721	19.0 (0.8)	19.5	18.7	19.6	0.9
**Sierra-Sacramento**	51,179	27.7 (15.5)	11.0	6.0	28.1	22.1
**Solano**	13,529	27.1 (4.4)	29.4	26.7	34.0	7.3
**Stanislaus**[a]	14,255	41.1 (7.0)	40.6	34.2	45.7	11.5
**Tuolumne**	2,500	10.7 (1.4)	10.0	10.0	11.5	1.5
**Ventura**	22,916	22.9 (4.6)	19.9	15.7	24.8	9.1
**Yolo**	7,734	46.3 (21.6)	41.5	30.7	78.8	48.1

Note: APOT-1 indicates 90th percentile offload time. EMS indicates emergency medical services. APOT-1 weighted mean, median, Q1, Q3, and IQR in minutes.
[a] Local EMS agencies with offload results missing for one or more months.

© 2024 Feldmeier et al. *JAMA Network Open*.

eTable 2. Monthly APOT-1 Weighted Means for the 5 Local EMS Agencies With the Highest and Lowest APOT-1 Weighted Means Over the Study Period

2a. Monthly APOT-1 Weighted Means for the 5 local EMS Agencies With the Highest APOT-1 Weighted Means Over the Study Period

Central California			Inland			Kern			Riverside			Sacramento		
**Month**	**Year**	**Weighted Mean (SD)**	**Month**	**Year**	**Weighted Mean (SD)**	**Month**	**Year**	**Weighted Mean (SD)**	**Month**	**Year**	**Weighted Mean (SD)**	**Month**	**Year**	**Weighted Mean (SD)**
January	2021	69.9 (20.2)	January	2021	58.8 (21.1)	January	2021	59.6 (7.5)	January	2021	63 (34.1)	January	2021	53.1 (14.9)
February	2021	60.5 (24.6)	February	2021	45 (17.6)	February	2021	48.1 (12.1)	February	2021	38.4 (15.8)	February	2021	45.1 (12.4)
March	2021	62.8 (23.7)	March	2021	43.9 (18)	March	2021	49.5 (11.7)	March	2021	33.9 (9.6)	March	2021	45.3 (13.7)
April	2021	68.1 (30.8)	April	2021	48.1 (20.7)	April	2021	46.5 (7.9)	April	2021	36.9 (12.3)	April	2021	51.6 (15)
May	2021	70 (32.8)	May	2021	48.5 (18.2)	May	2021	45.2 (6.6)	May	2021	38.8 (13.3)	May	2021	50.5 (14.9)
June	2021	71.7 (29.3)	June	2021	52.5 (23)	June	2021	45.3 (7.2)	June	2021	42.3 (16.7)	June	2021	59.6 (18.7)
July	2021	87.7 (65.7)	July	2021	57.1 (23.5)	July	2021	41.4 (22.9)	July	2021	49.8 (24.4)	July	2021	68.7 (16.8)
August	2021	103.2 (62.7)	August	2021	65.2 (27.1)	August	2021	64.8 (15)	August	2021	68.9 (30.7)	August	2021	85.5 (24.1)
September	2021	79.7 (37.9)	September	2021	64.5 (30.2)	September	2021	65.2 (14.9)	September	2021	61.2 (27)	September	2021	71.5 (25.2)
October	2021	76.6 (30.1)	October	2021	60.1 (26.6)	October	2021	55.5 (7.6)	October	2021	58.1 (24.7)	October	2021	70.8 (23.5)
November	2021	72 (25.6)	November	2021	60.9 (29.1)	November	2021	67.2 (14.6)	November	2021	59.1 (26.7)	November	2021	58.3 (19.7)
December	2021	59.2 (21.3)	December	2021	58.9 (20.5)	December	2021	53.3 (12.8)	December	2021	67.6 (31.4)	December	2021	74.6 (22.1)
January	2022	75.8 (26.6)	January	2022	72.4 (21)	January	2022	52.7 (17.2)	January	2022	93 (51.9)	January	2022	96.7 (33.6)
February	2022	53.7 (27.8)	February	2022	48.2 (14.6)	February	2022	43.3 (5.2)	February	2022	56.1 (25.7)	February	2022	55.2 (15.1)
March	2022	40.5 (10.5)	March	2022	45.5 (13.6)	March	2022	40.2 (5.5)	March	2022	45.3 (21.4)	March	2022	53.7 (12.3)
April	2022	39.7 (9.1)	April	2022	43.7 (14.7)	April	2022	44.8 (9)	April	2022	41.4 (17.9)	April	2022	51.1 (12.8)
May	2022	44.6 (8.4)	May	2022	46.7 (14.6)	May	2022	55.8 (13.9)	May	2022	41.4 (16.9)	May	2022	68.8 (24.6)
June	2022	44.3 (7.4)	June	2022	49.2 (16.1)	June	2022	56.1 (11.7)	June	2022	42.2 (18.9)	June	2022	79.7 (31.4)
July	2022	42.8 (6.3)	July	2022	48.1 (14.2)	July	2022	57 (11.8)	July	2022	48.4 (24.3)	July	2022	76.8 (28.4)
August	2022	40.2 (5.6)	August	2022	46.3 (14.4)	August	2022	56.5 (11.3)	August	2022	48.6 (24.2)	August	2022	58.6 (18.1)
September	2022	38.8 (5.4)	September	2022	45.6 (13)	September	2022	56.7 (11.7)	September	2022	46.7 (24.2)	September	2022	64.6 (17.9)
October	2022	36.8 (7.2)	October	2022	43.9 (12.5)	October	2022	59 (15.8)	October	2022	45.5 (21.6)	October	2022	61.8 (17.3)
November	2022	39.2 (10.2)	November	2022	46.7 (13.9)	November	2022	63.6 (12)	November	2022	59.2 (30.8)	November	2022	75.5 (18.2)
December	2022	44.6 (10.6)	December	2022	55.7 (16.8)	December	2022	64.6 (13.5)	December	2022	61.8 (35.7)	December	2022	84.8 (23.2)
January	2023	46.5 (7.9)	January	2023	49.4 (13.4)	January	2023	60.4 (13.1)	January	2023	49.5 (25.3)	January	2023	75.5 (23.7)
February	2023	45.6 (8.2)	February	2023	47.6 (13.3)	February	2023	59.4 (18.7)	February	2023	52.8 (26)	February	2023	75.1 (23.1)
March	2023	45.2 (6.5)	March	2023	46 (12.6)	March	2023	56.6 (15.1)	March	2023	49.5 (22.4)	March	2023	77.2 (19.5)
April	2023	43.7 (0)	April	2023	47.1 (0)	April	2023	53 (0)	April	2023	49.8 (0)	April	2023	67.8 (0)
May	2023	44.1 (0)	May	2023	46.6 (0)	May	2023	52.9 (0)	May	2023	43.2 (0)	May	2023	64.2 (0)
June	2023	44.4 (0)	June	2023	46.6 (0)	June	2023	52.4 (0)	June	2023	42.8 (0)	June	2023	69.1 (0)

Note: APOT-1 indicates 90th percentile offload time. EMS indicates emergency medical services. Only local EMS agencies with data for all 30 months of the study period were included. APOT-1 monthly weighted mean in minutes.

© 2024 Feldmeier et al. *JAMA Network Open*.

2b. Monthly APOT-1 Weighted Means for the 5 Local EMS Agencies With the Lowest APOT-1 Weighted Means Over the Study Period

San Benito			Tuolumne			Santa Barbara			Marin			North Coast		
**Month**	**Year**	**Weighted Mean (SD)**	**Month**	**Year**	**Weighted Mean (SD)**	**Month**	**Year**	**Weighted Mean (SD)**	**Month**	**Year**	**Weighted Mean (SD)**	**Month**	**Year**	**Weighted Mean (SD)**
January	2021	11.8 (0.7)	January	2021	8.2 (0)	January	2021	18.5 (1.9)	January	2021	13.7 (1.3)	January	2021	6.1 (2.8)
February	2021	12 (0.8)	February	2021	7 (0)	February	2021	17.7 (2.6)	February	2021	13.1 (0.9)	February	2021	5.8 (2.3)
March	2021	6.6 (0.3)	March	2021	6 (0)	March	2021	16.8 (0.8)	March	2021	13 (1.4)	March	2021	5.6 (2.6)
April	2021	3.9 (0.9)	April	2021	6 (0)	April	2021	13 (2.2)	April	2021	14.2 (1.3)	April	2021	5.6 (2.6)
May	2021	7.5 (0.6)	May	2021	7 (0)	May	2021	13.1 (1.6)	May	2021	12.7 (1.1)	May	2021	5.7 (2.6)
June	2021	3.1 (0.3)	June	2021	9 (0)	June	2021	12.5 (1.9)	June	2021	13.2 (1.5)	June	2021	5.5 (2.4)
July	2021	11.8 (1.6)	July	2021	8 (0)	July	2021	14 (1.9)	July	2021	12.9 (1.1)	July	2021	5.2 (3.1)
August	2021	11.7 (1.4)	August	2021	10 (0)	August	2021	13.5 (1.8)	August	2021	14.1 (0.9)	August	2021	5.3 (3.4)
September	2021	11.6 (1.1)	September	2021	8.9 (0)	September	2021	13.5 (0.2)	September	2021	13.4 (1.1)	September	2021	5.2 (3)
October	2021	10.7 (0.5)	October	2021	17.4 (0)	October	2021	12.7 (2)	October	2021	13.4 (1.4)	October	2021	5.3 (2.9)
November	2021	10.7 (0.6)	November	2021	18.1 (0)	November	2021	13.4 (2)	November	2021	13.3 (1.2)	November	2021	6 (3.2)
December	2021	10.7 (0.6)	December	2021	23.5 (0)	December	2021	12.8 (2.5)	December	2021	13.7 (0.4)	December	2021	6.5 (2.6)
January	2022	11.2 (1.7)	January	2022	0 (0)	January	2022	13.5 (2.7)	January	2022	13.6 (1.7)	January	2022	5.2 (3.1)
February	2022	11.4 (1.6)	February	2022	10 (0)	February	2022	13 (2.6)	February	2022	12.9 (1.1)	February	2022	5.3 (3.5)
March	2022	11.2 (1.4)	March	2022	10 (0)	March	2022	12.3 (2.9)	March	2022	14 (1.8)	March	2022	5.2 (3)
April	2022	10.1 (1.1)	April	2022	10 (0)	April	2022	13.1 (3.6)	April	2022	13.8 (0.8)	April	2022	6.7 (2.8)
May	2022	9.9 (0.6)	May	2022	10 (0)	May	2022	14 (2.9)	May	2022	14 (0.7)	May	2022	7.4 (3.1)
June	2022	9.9 (1.1)	June	2022	10 (0)	June	2022	12.6 (2.7)	June	2022	13.7 (1.5)	June	2022	6.8 (2.3)
July	2022	10 (2)	July	2022	10 (0)	July	2022	13.6 (2.7)	July	2022	13.5 (1.4)	July	2022	6.9 (3.1)
August	2022	10.3 (1.4)	August	2022	8 (0)	August	2022	14 (3.3)	August	2022	14.6 (0.9)	August	2022	6.4 (2.1)
September	2022	12.8 (2.6)	September	2022	9 (0)	September	2022	13 (3)	September	2022	14.5 (1.3)	September	2022	6.4 (1.7)
October	2022	9.4 (0.4)	October	2022	9.2 (1.1)	October	2022	13.1 (2.6)	October	2022	13.1 (1.5)	October	2022	6.2 (2)
November	2022	10.3 (1.5)	November	2022	10 (0.5)	November	2022	13.8 (2.4)	November	2022	13.2 (1.9)	November	2022	6.6 (2.1)
December	2022	11.3 (1.1)	December	2022	10 (0.3)	December	2022	13.4 (3)	December	2022	13.1 (1.5)	December	2022	6.9 (2.8)
January	2023	11.6 (1)	January	2023	10 (0)	January	2023	12.7 (2.7)	January	2023	13.1 (2)	January	2023	6.9 (3.1)
February	2023	10.4 (1.8)	February	2023	12 (0)	February	2023	12.5 (3)	February	2023	13 (1.5)	February	2023	6.9 (3.1)
March	2023	12 (1.2)	March	2023	10 (0)	March	2023	12.8 (3.4)	March	2023	12.9 (0.8)	March	2023	6.9 (3.4)
April	2023	11.5 (0)	April	2023	10 (0)	April	2023	13.6 (0)	April	2023	13 (0)	April	2023	7.4 (0)
May	2023	10.5 (0)	May	2023	9 (0)	May	2023	13 (0)	May	2023	13.6 (0)	May	2023	6.6 (0)
June	2023	13.7 (0)	June	2023	13 (0)	June	2023	12.9 (0)	June	2023	10.9 (0)	June	2023	7.4 (0)

Note: APOT-1 indicates 90th percentile offload time. EMS indicates emergency medical services. Only local EMS agencies with data for all 30 months of the study period were included. APOT-1 monthly weighted mean in minutes.

© 2024 Feldmeier et al. *JAMA Network Open*.
